# Red cell differential width (RDW) as a predictor of survival outcomes with palliative and adjuvant chemotherapy for metastatic penile cancer

**DOI:** 10.1007/s11255-020-02565-0

**Published:** 2020-07-23

**Authors:** Reena Patel, Louise English, Wing K. Liu, Alison C. Tree, Benjamin Ayres, Nick Watkin, Lisa M. Pickering, Mehran Afshar

**Affiliations:** 1 St George’s Medical School, University of London, London, UK; 2 Department of Urology, St George’s University Hospitals NHS Foundation Trust, London, UK; 3 Department of Oncology, St George’s University Hospitals NHS Foundation Trust, Blackshaw Road, Tooting, London, SW17 0QT UK; 4 The Royal Marsden NHS Foundation Trust Sutton, London, UK

**Keywords:** Penile cancer, Red cell differential width, Biomarker, Survival outcomes

## Abstract

**Purpose:**

Red cell distribution width (RDW) measures red cells’ size variability. Metastatic penile cancer displays poor chemotherapy response. As no validated prognostic predictor exists, we investigated whether RDW correlates independently with survival outcomes in metastatic penile cancer treated by chemotherapy.

**Methods:**

Electronic chemotherapy files of patients with metastatic penile cancer (M1 or N3) from a large academic supra-regional centre were retrospectively analysed between 2005 and 2018. Patients were stratified into RDW > 13.9% and < 13.9%, as per published data on RDW in renal cell carcinoma. Survival time was calculated from the date of chemotherapy initiation until the date of death.

**Results:**

58 patients were analysed. The RDW-high group (*n* = 31) had a poorer survival than the RDW-low group (*n* = 27). Median overall survival (mOS) in all patients was 19.0 months (95% CI 13.1–24.9). mOS for RDW-high was 15.0 months (95% CI 10.1–19.9) and 37.0 months (95% CI 32.3–43.1) for RDW-low. Kaplan–Meier curves showed a clear disparity in survival (log rank *p* = 0.025). Cox proportional hazard ratio for death, corrected for T-stage, grade, age and deprivation score was 0.43 (*p* = 0.04). Sub-analysis of the M1 patients showed mOS in RDW-high of 17 m (95% CI 11.6–22.4) vs. NR; HR for death of 0.42. N3 patients’ mOS in RDW-high cohort was 30 months (95% CI 4.5–55.9) vs. 13 months (95% CI 1.8–24.2) in RDW-low; HR for death was 0.30.

**Conclusion:**

RDW correlates independently with survival outcomes in metastatic penile cancer and may act as a potential predictor of survival outcomes for patients with metastatic penile cancer receiving chemotherapy.

## Introduction

Penile cancer is a rare disease with an incidence of less than 1 per 100,000 males in Western countries such as Europe and North America [1]. Metastatic penile cancer with spread to local lymph nodes has a 5-year survival rate of approximately 59% and an even poorer 5-year survival rate of 11% if spread to distant regions [2]. This poor prognosis is further compounded by the disease’s poor response rates to chemotherapy.

Tumour grade, as well as perineural and lymphatic invasion are important prognostic indicators of penile cancer. The presence of nodal involvement remains the most important indicator of survival outcome [3]. The method of obtaining these indicators via biopsy is invasive. Biomarkers in routine blood tests of cancer patients at time of diagnosis, as a tool in addition to conventional methods, may offer a minimally invasive cost-effective way of improving patient stratification into high and low risk subgroups to aid counselling and individualisation of patient care. To our knowledge, no validated baseline blood predictor exists for outcomes in stratifying patient management.

Red blood cell distribution width (RDW) is a widely available component of the full blood count that expresses the extent of the heterogeneity of erythrocyte size (anisocytosis) and is calculated as a coefficient of the mean corpuscular volume (MCV) [4]. RDW has conventionally been used in the investigation of anaemia. As a marker of inflammation and anaemia, RDW will naturally reflect the general condition of the patient, including age and other co-morbidities [5, 6], but recent studies have assessed its independent prognostic role and survival outcome in other solid and haematological cancers.

Numerous studies thereafter discussed different cut-off levels of RDW for patient stratification in cancer care. The selection of a 13.9% cutoff for RDW was established in recently published literature on renal cell carcinoma [7] and non-small cell lung cancer [8] to stratify high-risk and low-risk groups of cancer progression. Wang et al. first showed the correlation between RDW at the time of diagnosis and renal cell carcinoma stage and grade, defining advanced disease with a RDW cut-off value of 13.15% [9]. An RDW cut-off level of 14.15% and 14.5%, respectively, have been used as independent factors of poor survival in epithelial ovarian cancer [10] and chronic lymphocytic leukaemia [11].

Typically, factors such as age, performance status and tumour stage are used to stratify patients according to risk of cancer progression and, therefore, determine treatment decisions. Underpinned by a paucity of clinical research given the rarity of this disease, a lack of robust consensus on whether to give treatment in both palliative and adjuvant setting, and if so to who, necessitates identification of ways to determine which patients are more likely to benefit from therapy and survive longer. As RDW has been implicated in the systemic inflammation underlying neoplastic disease, we, therefore, hypothesized that RDW levels might be an easily accessible marker associated with progression and overall survival in patients with metastatic penile cancer receiving chemotherapy.

## Methods

The electronic medical records of 1777 patients with penile cancer in a large academic supra-regional centre for penile cancer were retrospectively analysed, with dates of referral ranging from June 2001 to March 2018. From this large dataset, 75 patients with a diagnosis of metastatic penile cancer—defined by our group as either M1 disease or N3 disease, receiving palliative or adjuvant chemotherapy, were identified. A further 17 patients were excluded as the data on date of diagnosis or baseline haematology results were not available. The final cohort included 58 patients diagnosed between April 2005 and March 2018. The diagnosis of penile cancer was confirmed pathologically with biopsy.

The following variables were included to assess the prognostic role of RDW: age at time of diagnosis, chemotherapy regimen, histopathology, staging, date of progression, date of death, baseline and pre-cycle full blood count. In addition, patient deprivation scores from the national Index of Multiple Deprivation were collected. Patients were divided into two groups according to a baseline (first blood result after referral) RDW cut-off value established in previously discussed published renal cell carcinoma data [11]: RDW-low (< 13.9%) and RDW-high (> 13.9%). The reference range for normal RDW in St George’s University Hospital laboratory is 11.5–15.0%. Survival time was calculated from the date of chemotherapy initiation until the date of death or the date of the last consultation. Statistical analysis was carried out using SPSS Ver. 25 Software. Cox regression analysis was performed to assess hazard ratios for death and results were corrected for stage, grade, age, anaemia and social deprivation score. The Kaplan Meier method was used to analyse the relationship between the two RDW group’s survival times with log rank testing.

## Results

58 patients were analysed post exclusion. 43 were M1 (metastatic disease to distant sites outside of the pelvic lymph nodes) and 15 were N3 (positive pelvic lymph node disease—who received adjuvant chemotherapy). Median follow-up was for 449 days (interquartile range 187–797). Follow-up was for a maximum of 3275 days (9 years). 30 (51.7%) patients died during this follow-up period. 11 patients from the RDW-low group (40.7%) and 19 patients from the RDW-high group (61.3%) died. Patient demographics, chemotherapy regimens and histopathology of the low and high RDW subgroups are displayed in Table 1. Table 2 shows components of patients’ pre-treatment full blood count.

**Table 1:** Baseline patient demographics, chemotherapy regimes and histopathology stratified according to RDW level

	RDW-low (< 13.9%; *n* = 27), *n* (%)	RDW-high (> 13.9%; *n* = 31), *n* (%)
Median age (range)	57 (31–83)	63 (43–80)
Socioeconomic status (IMD quintiles)		
I	2 (3.4%)	4 (6.9%)
II	8 (29.6%)	9 (29.0%)
III	5 (18.5%)	2 (6.5%)
IV	7 (25.9%)	6 (19.4%)
V	3 (11.1%)	10 (32.3%)
Other	2 (7.4%)	0 (0.0%)
First-line therapy		
Cisplatin + capecitabine	10 (17.2%)	15 (25.9%)
VinCaP (vinflunine)	1 (1.7%)	1 (1.7%)
TIP (paclitaxel + isosfamide + cisplatin)	0 (0.0%)	1 (1.7%)
Carboplatin + methotrexate + bleomycin	0 (0.0%)	1 (1.7%)
TPF (docetaxel + cisplatin + 5-FU)	2 (3.4%)	1 (1.7%)
Other	14 (24.1%)	12 (20.7%)
pT stage		
1	5 (18.5%)	5 (16.1%)
2	13 (48.1%)	11 (35.5%)
3	8 (29.6%)	10 (32.3%)
4	1 (3.7%)	2 (6.5%)
Unknown	0 (0.0%)	3 (9.7%)
pN stage		
0	2 (7.4%)	2 (6.5%)
1	5 (18.5%)	4 (12.9%)
2	6 (22.2%)	8 (25.8%)
3	5 (18.5%)	5 (16.1%)
Unknown	9 (33.3%)	12 (38.7%)
Grade		
1	0 (0.0%)	0 (0.0%)
2	4 (14.8%)	5 (16.1%)
3	23 (85.2%)	22 (71.0%)
Other	0 (0.0%)	4 (13.0%)

**Table 2:** Pre-treatment full blood count

	RDW-low (< 13.9%; *n* = 27), median (range)	RDW-high (> 13.9%; *n* = 31), median (range)
Haemoglobin (g/L)	141 (104–163)	112 (77–152)
Platelets (10^9^/L)	298 (68–492)	303 (176–817)
Neutrophils (10^9^/L)	5.7 (1.8–12.8)	6.4 (2.1–21.4)
Lymphocytes (10^9^/L)	2.0 (1.0–3.2)	1.2 (0.4–2.4)
RDW (%)	12.9 (11.8–13.8)	14.7 (13.9–23.9)

**Fig. 1:**
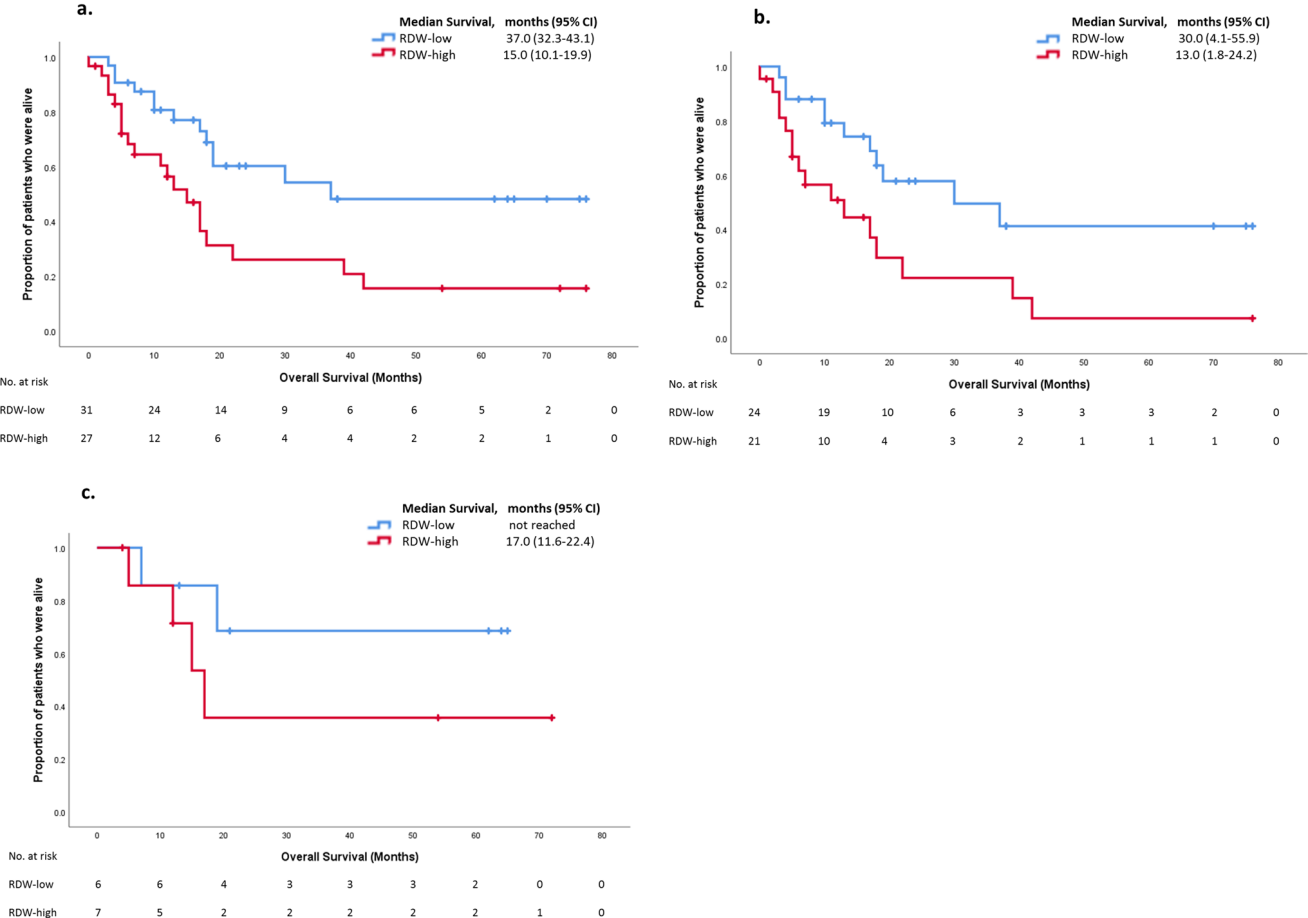
**a** Kaplan-Meier plot of median overall survival between RDW-high and RDW-low groups for all patients. **b** Kaplan-Meier plot of median overall survival between RDW-high and RDW-low groups for patients receiving adjuvant chemotherapy. **c** Kaplan-Meier plot of median overall survival between RDW-high and RDW-low groups for patients receiving palliative chemotherapy

Patients in the RDW-high group (*n* = 31) had a poorer survival than the RDW-low group (*n* = 27) (Fig. 1a). Median overall survival (mOS) in the whole group was 19.0 months (95% CI 13.1–24.9). For RDW-high mOS was 15.0 months (95% CI 10.1–19.9) and for RDW-low it was 37.0 months (95% CI 32.3–43.1). Kaplan–Meier curves show a clear disparity in survival (log rank *p* = 0.025), and Cox proportional hazard ratio for death, corrected for T-stage, grade, age, anaemia and deprivation score was 0.43 (*p* = 0.04) (Fig. 1a). When patients were sub-analysed, those receiving adjuvant chemotherapy had a mOS in the RDW-high cohort of 30 months (95% CI 4.1–55.9), vs. a mOS in the RDW-low cohort of 13 months (95% CI 1.8–24.2) (Fig. 1b). The hazard ratio for death in this cohort was 0.30. In the palliative chemotherapy group, mOS in the RDW-high cohort was 17 months (95% CI 11.6–22.4), with those in the RDW-low cohort not reaching median (NR) (Fig. 1c). Hazard ratio for death, when corrected for T-stage, grade, age, anaemia and deprivation score was 0.42.

## Discussion

To our knowledge, this is the first work done on the association between RDW and survival outcomes in patients with metastatic penile cancer. The key finding is that in metastatic penile cancer, patients with a high RDW value before initiation of systemic therapy were associated with advanced disease and, therefore, had a poorer survival than patients with low RDW values. We took steps to address the potential confounding effects of higher stage, grade and lower haemoglobin in the poorer prognosis group, by stratifying our patients in the Cox regression analyses to obtain hazard ratios for death corrected for stage, grade, age, anaemia and socio-economic deprivation, thus identifying RDW as an independent biomarker in this setting.

Work on prognostic indicators in penile cancer has added to the way in which patients can be stratified and managed. High levels of RDW and other inflammatory markers have been implicated with aggressive cancers that show poor chemotherapy response. Similar results were noted in renal cell carcinoma treated with partial and radial nephrectomy according to an RDW cut-off of 13.9%, concluding that pre-operative levels can aid in assessing the tumour aggressiveness [7]. The use of RDW as a prognostic indicator in cancer has been re-iterated in a meta-analysis of 17 studies across a number of tumour types, including lung and oesophageal cancer [12]. Furthermore, the Tromso Study demonstrated that elevated pre-treatment levels correlated to unfavourable pathological prognostic factors, such as tumour size, grade and lymph node involvement [13]. They found that a small 1% increase in RDW was associated with a significant rise in the risk of regional cancer spread by 21% and distal metastases by 19% [13]. Interestingly, RDW could possibly be monitored for changes after several cycles of chemotherapy to assess for treatment response [14].

Systemic inflammation plays an important role in the progression of cancer, with emerging evidence that simple inflammatory markers in the full blood count can aid clinicians in targeting and monitoring treatment. Pre-treatment neutrophil–lymphocyte ratio (NLR), which uses two readily available components of the full blood count, has extensively been investigated as an independent prognostic indicator of poor survival in other solid urological malignancies, such as renal cell carcinoma and upper tract urothelial carcinoma [15–17]. In addition, a high pre-treatment CRP value greater than 15 mg/L was associated with advanced staging, greater metastatic spread and a poorer 5-year survival outcome in penile cancer [18]. However, their use in clinical practice is limited when monitoring a patient’s cancer progression, or indeed their baseline risk of death.

The underlying mechanism between RDW and cancer progression is not clearly understood. A high RDW may be driven by the excessive production of cytokines in the tumour microenvironment, such as IL-6 and TNF-a [19, 20]. However, it is difficult to clarify whether a high RDW level is due to the systemic inflammatory process or the poor chemotherapy response [21]. Chronic inflammation impairs iron metabolism and elevated RDW may be closely associated with inadequate erythropoeisis as part of the natural ageing process amongst cancer patients [22]. A high RDW level in cancer patients may also be associated with anaemia and poor nutritional status. In addition, RDW has been implicated with poor survival outcomes in patients showing signs of cachexia [21, 23, 24].

Due to the low incidence of the disease and the specificity of our inclusion criteria, one of the main limitations is that the study findings can only apply to patients with metastatic penile cancer receiving palliative or adjuvant chemotherapy and, therefore, cannot be used in the stratification of patients undergoing other forms of cancer therapy, such as surgery and radiotherapy. Furthermore, a significant proportion of patients were excluded from this analysis due to a lack of sufficient baseline laboratory results. Moreover, RDW was not adjusted for confounding factors such as cardiovascular disease and infections that may influence the RDW level. We did, however, take steps in correcting our findings for a number of other potential confounders. As an important next step, we would recommend validation of the findings of the present study in a separate cohort.

## Conclusion

As penile cancer is rare, there is little data available on biomarkers of response to aid individualisation of patient therapy. Our data suggests that high pre-treatment RDW can be used as a potential predictor of unfavourable survival outcomes for patients with metastatic penile cancer receiving chemotherapy. This inexpensive widely-available blood marker of inflammation could possibly be used in the clinical setting to stratify patients into high and low risk groups of metastatic penile cancer progression and, therefore, aid in the decision-making process regarding their treatment pathway; however, further validation is required. This finding also raises the possibility that RDW normalisation could be used as a tool to assess the efficacy of chemotherapy cycles in penile cancer, and this is an area warranting further investigation.

## Data Availability

Data not available. Due to the sensitive nature of the data in this study, raw data would remain confidential and would not be publicly available.
